# *StSAUR31* functions as a negative regulator of enzymatic browning in potato (*Solanum tuberosum* L.)

**DOI:** 10.1093/hr/uhag115

**Published:** 2026-04-10

**Authors:** Zan Meng, Miaomiao Zhang, Yurong Ma, Guangcun Li, Shiyang Liu, Yu Cao, Yujie Niu, Qingqing Li, Qingguo Wang

**Affiliations:** College of Food Science and Engineering, Shandong Agricultural University, Tai’an 271018, Shandong, China; College of Food Science and Engineering, Shandong Agricultural University, Tai’an 271018, Shandong, China; School of Life Sciences, Yantai University, Yantai 264005, Shandong, China; State Key Laboratory of Vegetable Biobreeding, Key Laboratory of Biology and Genetic Improvement of Tuber and Root Crop of Ministry of Agriculture and Rural Affairs, Institute of Vegetables and Flowers, Chinese Academy of Agricultural Sciences, Beijing 100081, China; College of Food Science and Engineering, Shandong Agricultural University, Tai’an 271018, Shandong, China; Postharvest Lab, Shandong Provincial Research Centre for the Engineering and Technology of Food Safety of Fruit and Vegetables, Jinan 251499, Shandong, China; College of Food Science and Engineering, Shandong Agricultural University, Tai’an 271018, Shandong, China; College of Food Science and Engineering, Shandong Agricultural University, Tai’an 271018, Shandong, China; College of Food Science and Engineering, Shandong Agricultural University, Tai’an 271018, Shandong, China; College of Food Science and Engineering, Shandong Agricultural University, Tai’an 271018, Shandong, China

## Abstract

Enzymatic browning significantly affects the processing and quality maintenance of a wide range of horticultural produce. Identifying key regulators of browning is essential for elucidating its underlying mechanisms and developing effective mitigation strategies. In this study, transcriptomic comparison between potato cultivars with contrasting browning sensitivities identified a *small auxin-up RNA*, *StSAUR31*, as a potential regulator of auxin-mediated browning inhibition in potato. Functional analyses showed that overexpression of *StSAUR31* markedly reduced browning intensity and PPO activity, whereas knockout of *StSAUR31* produced the opposite phenotype. Correspondingly, StuPPO1 protein abundance decreased in *StSAUR31* overexpression lines and increased in knockout lines. Mechanistically, StSAUR31 physically interacted with StuPPO1 in an auxin-enhanced manner, partially altering its subcellular localization and reducing its accumulation in plastids. Additionally, *StSAUR31* downregulated *StuPPO1* expression, reduced endogenous free tyrosine levels, and enhanced antioxidant capacity. Collectively, these findings indicated that *StSAUR31* coordinately regulated PPO activity, substrate availability, and antioxidant capacity, thereby integrating multiple mechanisms to suppress enzymatic browning in potatoes. These results advance our understanding of the crosstalk between auxin signaling and enzymatic browning, providing new insights into the role of hormone signaling in postharvest quality regulation.

## Introduction

Enzymatic browning is a major challenge affecting the visual quality and market value of horticultural crops [1]. This process is primarily catalyzed by polyphenol oxidase (PPO), a nucleus-encoded enzyme that oxidizes phenolic substrates into brown pigments. Substantial progress has been made in suppressing enzymatic browning through genetic manipulation of *PPO* expression, such as CRISPR/Cas9-mediated gene editing and RNA interference (RNAi), which primarily target *PPO* at the transcriptional level [2–5]. However, the post-translational regulation of PPO remains poorly understood. PPOs are synthesized on cytosolic ribosomes as inactive precursors and subsequently imported into plastids, where they undergo proteolytic processing to become catalytically active under stress conditions [6–8]. While plastid import and processing are essential for the maturation of plastid-localized proteins, increasing evidence suggests that these proteins can be regulated in the cytosol before plastid translocation [9, 10]. However, whether and how PPOs are regulated during their cytosolic stage remains largely unknown.

Plant hormones such as auxin, jasmonic acid (JA), salicylic acid (SA), abscisic acid (ABA), and ethylene play pivotal roles in mediating plant responses to abiotic and biotic stresses, including wounding, drought, pathogen attack, and oxidative stress [11]. These hormones have also been implicated in the regulation of PPO activity, although their regulatory effects appear to differ. SA and JA have been shown to promote PPO activation. For instance, methyl salicylate treatment induced *PPO* transcription and promoted the import of pPPO into chloroplasts, where it is targeted to the thylakoid [12]. Similarly, methyl jasmonate treatment significantly enhanced pPPO import into chloroplasts in tomato and tobacco [13], suggesting that SA and JA actively promoted PPO activation at both transcriptional and post-translational levels. In contrast, exogenous indole-3-acetic acid (IAA) application has been shown to reduce PPO activity and alleviate browning in mango and royal date [14, 15]. However, whether auxin suppresses enzymatic browning in potatoes remains unclear. In potato, nine *PPO* genes (*StuPPO1–9*) have been identified, among which *StuPPO1*, *StuPPO2*, and *StuPPO3* are the predominant isoforms expressed in tubers and contribute significantly to tuber browning [3]. Despite these advances, the molecular mechanisms linking auxin signaling to *PPO* regulation remain largely unexplored.

*Small auxin-up RNAs* (*SAURs*) constitute a large family of auxin-responsive genes, with the transcription being robustly induced by auxin and mediating diverse auxin-regulated physiological processes [16]. Members of the *SAUR* family respond to a range of environmental stresses, including insect attack, light exposure, drought, and salinity [17, 18]. Increasing evidence suggests that *SAURs* function as signaling hubs that integrate various hormonal and environmental signals [19]. For example, *Arabidopsis AtSAUR41* and wheat *TaSAUR78* have been reported to positively regulate plant responses to drought, salinity, and temperature extremes [20, 21]. In addition, certain *SAUR* genes are induced under tissue injury or pathogen-associated conditions, such as *StSAUR-AC1* during cutting-induced stress [22], and *OsSAUR22*, *OsSAUR25*, and *OsSAUR53* during *Striga hermonthica* infection [23]. Notably, several *SAUR* genes exhibit differential expression during callus formation and tissue regeneration [24], processes that are typically accompanied by oxidative stress and enzymatic browning. However, whether *SAURs* are involved in the regulation of enzymatic browning remains unknown.

In this study, transcriptome analysis between two potato cultivars with distinct browning susceptibility identified *StSAUR31* as a potential regulator of enzymatic browning in potato. We generated *StSAUR31* overexpression and knockout tubers for functional characterization. Results demonstrated that *StSAUR31* negatively regulated PPO activity and enzymatic browning in potatoes. Mechanistically, *StSAUR31* suppressed browning by coordinately regulating StuPPO1 abundance and subcellular localization, substrate availability, and antioxidant capacity. Our findings provide new insights into the auxin-mediated regulatory mechanism of enzymatic browning and offer potential molecular targets for improving the postharvest quality of horticultural produce.

## Results

### Identification of *StSAUR31* as a candidate regulator of auxin-mediated browning inhibition in potatoes

To elucidate the molecular basis underlying cultivar-dependent sensitivity to enzymatic browning, we analyzed the changes in visual browning and PPO activity following tuber cutting in cultivars ‘K4’ (browning-sensitive) and ‘K13’ (browning-resistant). Both parameters were significantly lower in ‘K13’ than ‘K4’ during storage, with the greatest differences observed at 3 d (Fig. S1). To identify key genes involved in regulating browning, we therefore analyzed the transcriptome data of these two cultivars at 0 d and 3 d after cutting from our previous studies [25, 26]. Gene Ontology (GO) enrichment analysis revealed significant enrichment of hormone response terms, suggesting a potential role of hormonal signaling in regulating browning responses (Fig. S2). Notably, several *SAUR* genes exhibited distinct and cultivar-dependent expression patterns. In ‘K4,’ four *SAUR* genes (*StSAUR36*, *StSAUR44*, *StSAUR71-1*, and *StSAUR71-2*) exhibited significant transcriptional changes in response to cutting (Table S1), whereas in ‘K13,’ three *SAUR* genes (*StSAUR36*, *StSAUR44*, and *StSAUR31*) were regulated by cutting (Table 1; Table S1). The expression responses of these auxin-related genes to cutting implied a potential role of auxin signaling in modulating cultivar-dependent browning differences.

**Table 1:** Fold changes of St*SAUR31* gene in potato tubers of ‘K4’ and ‘K13’ after cutting

Gene ID	Annotation	Fold change (‘K4’-3 d vs ‘K4’-0 d)	Fold change (‘K13’-3 d vs ‘K13’-0 d)
*PGSC0003DMG400030231*	*StSAUR31*	No change	1.56

**Figure 1:**
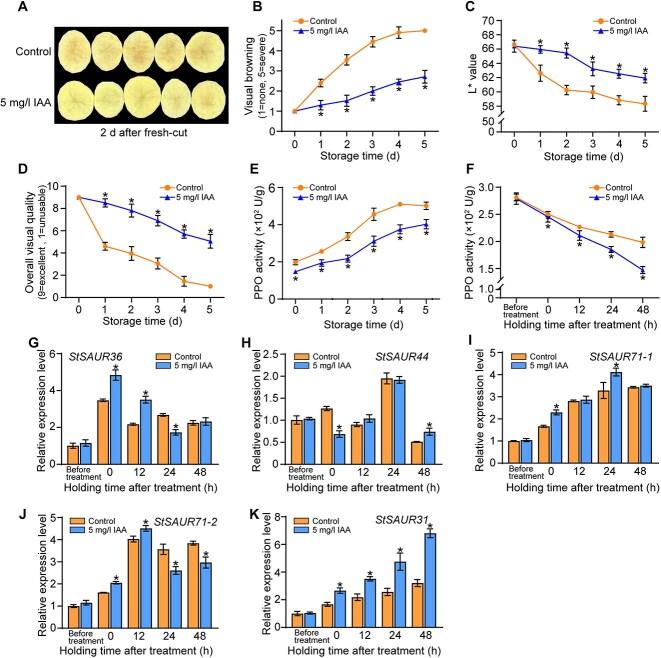
Pre-cutting IAA treatment inhibits the browning of fresh-cut potatoes. (A) Representative images of fresh-cut potato slices on 2 d after IAA treatment. (B) Visual browning. (C) Lightness (L^*^) value. (D) Overall visual quality. (E and F) PPO activity. (G–K) Relative expression levels of *StSAUR* genes after IAA treatment. For Fig. 1F–K, the ‘0 h’ on the *x*-axis represents samples collected immediately after the 10-min of IAA treatment. The asterisks indicate significant differences (^*^P < 0.05) as determined by Student’s *t*-test.

To investigate the potential inhibitory effect of auxin on browning, fresh-cut potato slices of the cultivar ‘Desiree’ were treated with different concentrations of IAA (0, 1, 5, 25, and 100 mg/l). Direct application of IAA to fresh-cut potato slices resulted in limited browning inhibition, as only 25 and 100 mg/l IAA produced slight reduction in browning (Fig. S3). However, pre-cutting treatment of intact tubers with IAA followed by holding at 25°C for 12–72 h markedly alleviated the browning of fresh-cut potatoes (Table S2). The strongest inhibition was observed when tubers were treated with 5 mg/l IAA for 10 min, followed by a 48-h holding period before cutting (Table S2). Relative to the control, pre-cutting IAA treatment at 5 mg/l reduced browning and maintained higher lightness (L^*^) values (Fig. 1A–C). The control lost marketable quality on 1 d (Overall visual quality score < 6), whereas IAA-treated slices remained acceptable for up to 4 d (Fig. 1D), indicating that pre-cutting IAA treatment extended the shelf life of fresh-cut potatoes by at least 3 d.

Consistent with the visual phenotype, PPO activity was significantly decreased after IAA treatment (Fig. 1E), with notable differences already observed at 0 d (samples treated with 5 mg/l IAA for 10 min and then held at 25°C for 48 h). PPO activity declined progressively during the pre-cutting holding period and reached the maximum difference from the control at 48 h (Fig. 1F). Similar trends were also observed in the cultivars ‘K4’ and ‘Netherland 15,’ both showing markedly lower PPO activities under the same pre-cutting IAA treatment condition (Fig. S4). These results indicated that IAA-mediated browning inhibition was consistent across cultivars and closely associated with reduced PPO activity. To investigate whether auxin-mediated browning inhibition was associated with *SAUR* gene expression, the transcript levels of the five *SAUR* genes were examined during the 48 h holding period following 5 mg/l IAA treatment (Fig. 1G–K). Compared with the control, *StSAUR36* was upregulated within 12 h and subsequently downregulated (Fig. 1G), whereas *StSAUR44* was slightly upregulated at 48 h after IAA treatment (Fig. 1H). *StSAUR71-1* showed little difference between control and IAA treatment (Fig. 1I), while *StSAUR71-2* upregulated within 12 h but downregulated after 24 h after IAA treatment (Fig. 1J). In contrast, *StSAUR31* displayed a rapid induction after IAA treatment, remaining consistently higher than the control and peaking at 48 h (Fig. 1K). This expression pattern was opposite to the changes of PPO activity (Fig. 1F). These results suggested that *StSAUR31* may act as a key regulator of auxin-mediated browning inhibition in potatoes.

### *StSAUR31* negatively regulates enzymatic browning in potatoes

**Figure 2:**
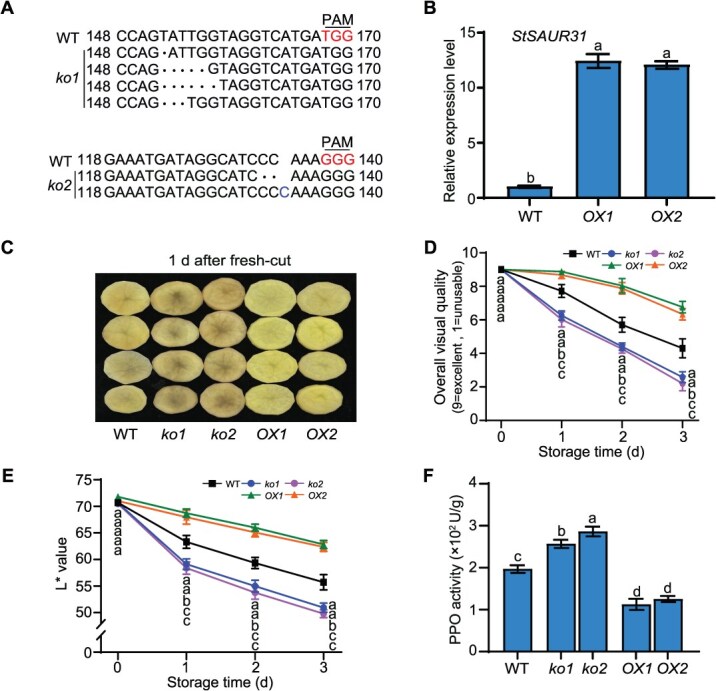
Functional characterization of *StSAUR31* in regulating enzymatic browning in potatoes. (A) Next-generation sequencing showing deletions or insertions in the target region in two knockout lines (*ko1* and *ko2*). (B) qRT-PCR analysis of *StSAUR31* expression in WT and *StSAUR31* overexpression lines *(OX1* and *OX2)*. (C) The browning phenotypes of WT and *StSAUR31* transgenic tubers at 1 d after fresh-cut. (D) Overall visual quality of potato slices during storage. (E) Lightness (L^*^) values of potato slices during storage. (F) PPO activity at 0 d (samples collected immediately after cutting). Different letters indicate significant differences (*P* < 0.05) as determined by LSD test.

To explore the functional role of *StSAUR31* in enzymatic browning, we generated overexpression (*OX*) and CRISPR/Cas9-mediated knockout (*ko*) lines. Two independent knockout lines (*ko1* and *ko2*) were obtained using two guide RNAs targeting different regions of the coding sequence (Fig. S5). Next-generation sequencing confirmed successful editing at the target sites, resulting in deletions or insertions that disrupted the *StSAUR31* open reading frame (Fig. 2A). Transgenic lines overexpressing *StSAUR31* showed significantly elevated transcript levels compared to wild type (WT) (Fig. 2B). Phenotypic analysis revealed that potato slices from *OX* lines showed noticeably reduced browning, while those from *ko* lines exhibited accelerated browning compared to WT (Fig. 2C). The browning differences among genotypes were most distinct at 1 d after fresh-cut, with WT tubers showing moderate browning, *ko* lines exhibiting severe browning approaching the commercial acceptability threshold (overall visual quality score ~6), whereas *OX* lines remaining largely unbrowned (Fig. 2C and D). The overall visual quality and L^*^ values were significantly higher in *OX* lines and lower in *ko* lines during storage (Fig. 2D and E). As the key enzyme mediating enzymatic browning, PPO activity increased by 32.18% and 40.41% in the two knockout lines, whereas it decreased by 42.81% and 36.30% in the two overexpression lines compared with WT (Fig. 2F), indicating that *StSAUR31* inhibited enzymatic browning by suppressing PPO activity.

### Proteomic insights into *StSAUR31*-mediated browning regulation

**Figure 3:**
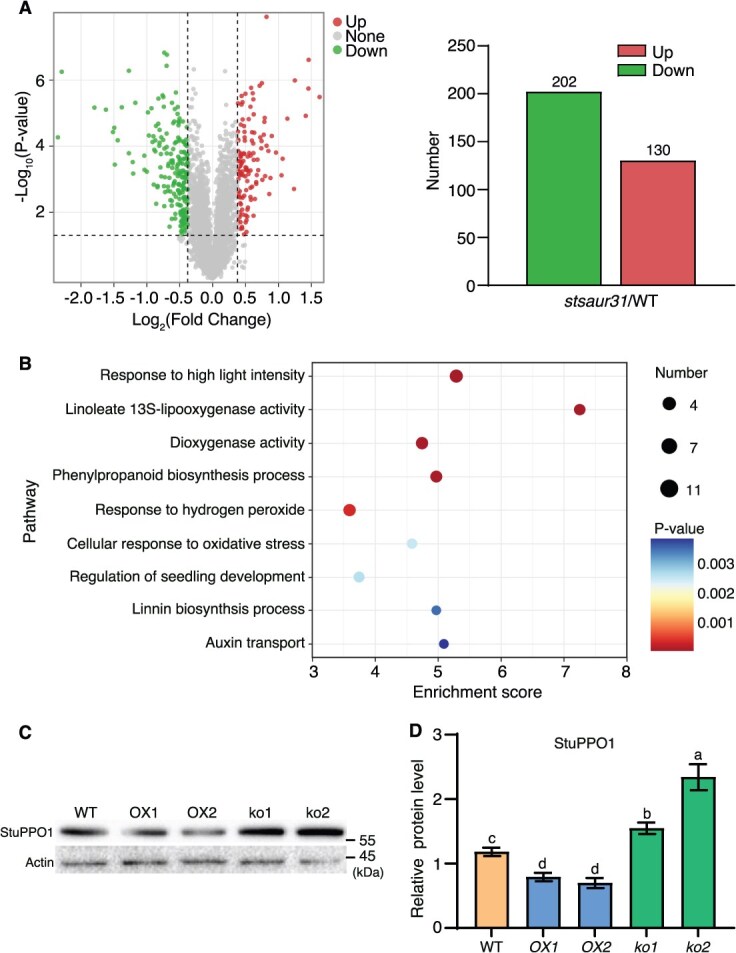
Proteomic analysis of DAPs and western blot validation of StuPPO1 in *StSAUR31* transgenic lines. (A) Analysis of DAPs between WT and *stsaur31* knockout lines. Significantly upregulated and downregulated proteins are indicated. (B) GO enrichment analysis of DAPs in WT and *stsaur31* knockout tubers. (C) StuPPO1 protein abundance in potato tubers by western blotting. (D) Relative StuPPO1 protein levels were quantified by ImageJ. Values represent mean ± SD from three biological replicates. Different letters indicate significant differences at *P* < 0.05 (one-way ANOVA).

To further explore the potential mechanism by which *StSAUR31* regulated enzymatic browning, TMT-based quantitative proteomics analysis was conducted between WT and *stsaur31* knockout tubers (Table S3). The volcano plot analysis revealed a total of 332 differentially accumulated proteins (DAPs), including 130 upregulated and 202 downregulated proteins in *stsaur31* versus WT (Fig. 3A). GO enrichment analysis revealed significant enrichment in pathways related to linoleate 13S-lipoxygenase activity, dioxygenase activity, response to hydrogen peroxide, phenylpropanoid and lignin biosynthetic processes, and cellular response to oxidative stress (Fig. 3B). These enriched pathways are closely linked to oxidative metabolism and phenolic compound oxidation, which constitute key biochemical processes underlying enzymatic browning. Notably, the enrichment of auxin transport and regulation of seedling development categories suggested that *StSAUR31* may alter auxin signaling and distribution. We observed that overexpression of *StSAUR31* increased IAA levels and upregulated the expression of *StYUCCA6*, *StYUCCCA8*, and *StYUCCA10*. Knockout of *StSAUR31* exhibited decreased IAA content and reduced *YUCCA* genes expression (Fig. S6), suggesting that *StSAUR31* may regulate auxin levels by modulating *YUCCA* genes expression.

As a key enzyme mediating enzymatic browning in potato, StuPPO1 showed significantly increased abundance in the *stsaur31* knockout mutant based on the proteomic analysis (Table S3). To validate these results, a StuPPO1-specific antibody was generated and used to examine its protein abundance in WT and *StSAUR31* transgenic tubers by western blot (Fig. 3C). The results showed that StuPPO1 protein levels were on average 36.99% lower in *StSAUR31* overexpression lines and 64.58% higher in *stsaur31* knockout mutant compared with WT (Fig. 3D). These resultsd indicated that *StSAUR31* negatively regulated StuPPO1 accumulation.

### StSAUR31 interacts with StuPPO1 in an auxin-enhanced manner

**Figure 4:**
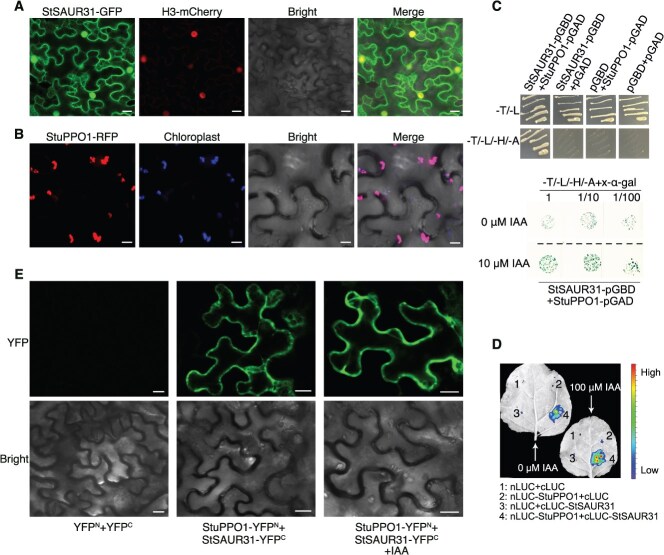
StSAUR31 directly interacts with StuPPO1. (A) Subcellular localization of StSAUR31 in *N. benthamiana*. Scale bar, 20 μm. (B) Subcellular localization of StuPPO1 in *N. benthamiana*. Scale bar, 10 μm. (C) Y2H assay showing the interaction between StSAUR31 and StuPPO1. (D) LCI assay demonstrating the binding of StSAUR31 to StuPPO1. (E) BiFC assay confirming the interaction of StSAUR31 with StuPPO1. Scale bar, 20 μm.

To elucidate how *StSAUR31* regulated StuPPO1 at the protein level, we first examined their subcellular localization. StSAUR31 was located in both the nuclei and cytoplasm (Fig. 4A). StuPPO1 was localized in the chloroplast as normally recognized (Fig. 4B). Yeast two-hybrid (Y2H) assays confirmed a direct interaction between StSAUR31 and StuPPO1 (Fig. 4C). To validate this interaction in planta, luciferase complementation imaging (LCI) was performed in *Nicotiana benthamiana* leaves. A strong luminescence signal was detected when StSAUR31 and StuPPO1 were co-expressed (Fig. 4D). Consistently, bimolecular fluorescence complementation (BiFC) assays showed a reconstituted YFP signal in the cytoplasm (Fig. 4E), indicating that the interaction occurred predominantly in the cytosolic compartment. Notably, exogenous IAA treatment enhanced the interaction between StSAUR31 and StuPPO1 in both yeast and tobacco systems (Fig. 4C–E). These results demonstrated that StSAUR31 directly interacted with StuPPO1 in an auxin-enhanced manner.

### StSAUR31 alters the subcellular localization of StuPPO1

**Figure 5:**
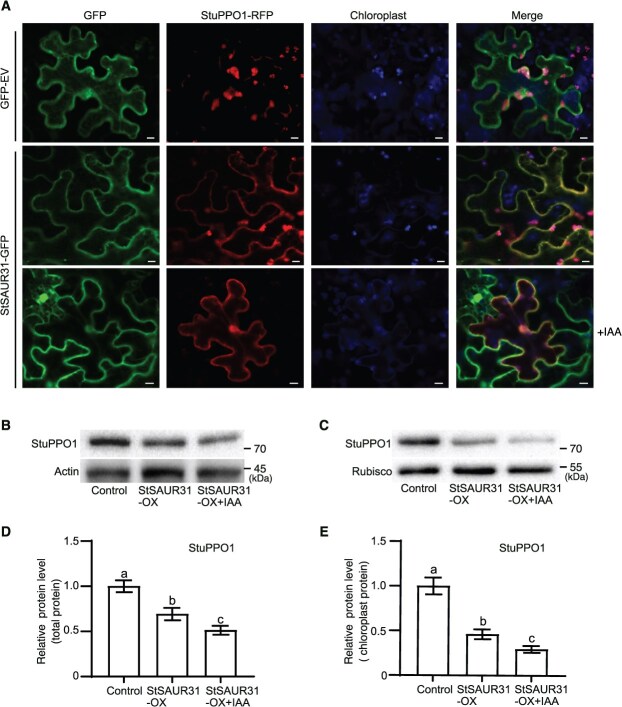
StSAUR31 alters the subcellular localization and plastid accumulation of StuPPO1 in *N. benthamiana*. (A) Subcellular localization of StuPPO1-RFP co-expressed with empty GFP (control) or StSAUR31-GFP in tobacco leaves. Scale bar, 10 μm. (B and C) Immunoblot analysis of StuPPO1 in total (B) and chloroplast protein (C) from control and *StSAUR31*-overexpressing tobacco leaves. (D and E) Relative StuPPO1 levels in total (D) and chloroplast protein (E). Values represent mean ± SD from three biological replicates. Different letters indicate significant differences at *P* < 0.05 (one-way ANOVA).

To investigate whether StSAUR31 affected the subcellular localization of StuPPO1, StSAUR31-GFP driven by the *CaMV 35S* promoter and StuPPO1-RFP were transiently co-expressed in *N. benthamiana* leaves. As shown in Fig. 5A, in the control leaves expressing StuPPO1-RFP with empty GFP, the red fluorescence was mainly localized to chloroplasts. In contrast, transient overexpression of *StSAUR31* partially altered the subcellular localization of StuPPO1, resulting in increased cytoplasmic localization (Fig. 5A). Quantitative analysis confirmed that overexpression of *StSAUR31* reduced plastid-localized StuPPO1-RFP fluorescence intensity, and this reduction was further enhanced by IAA treatment (Fig. S7). To further validate whether *StSAUR31* affected the subcellular localization of StuPPO1 in a homologous system, we isolated protoplasts from the leaves of WT and *StSAUR31*-*OX* lines and transiently expressed the StuPPO1-GFP fusion protein (Fig. S8). Results showed that StuPPO1-GFP fluorescence was predominantly localized in chloroplasts in WT protoplasts. In contrast, in the *StSAUR31-OX* background, the chloroplast localization of StuPPO1-GFP was partially altered, and additional fluorescence signals were observed in the cytoplasm (Fig. S8). The results demonstrated that overexpression of *StSAUR31* partially altered the subcellular localization of StuPPO1, which was consistent with the results obtained in *N. benthamiana* leaves.

To validate this observation, total and chloroplast proteins were isolated from *N. benthamiana* leaves transiently expressing StuPPO1 alone or co-expressing it with StSAUR31, and analyzed by immunoblotting with an anti-StuPPO1 antibody (Fig. 5B and C). Actin and Rubisco were used as reference proteins for total and chloroplast proteins, respectively. The consistent abundance of Rubisco levels across samples confirmed its suitability as a reference protein for chloroplast proteins (Fig. S9). Western blot analysis revealed that overexpression of *StSAUR31* reduced total StuPPO1 abundance by 30.81%, whereas chloroplast-localized StuPPO1 decreased by 53.84% (Fig. 5D and E). Exogenous IAA treatment further enhanced these reductions to 48.71% and 70.41%, respectively. When the chloroplast localization efficiency of StuPPO1 (defined as the ratio of chloroplast StuPPO1 to total StuPPO1) in the control was set as 100%, *StSAUR31* overexpression reduced this value to 66.71%, and it further decreased to 57.70% after IAA treatment (Fig. S10). Together, these results indicated that StSAUR31 modulated the subcellular localization of StuPPO1, contributing to reduced plastid accumulation.

## Discussion

Enzymatic browning, primarily driven by PPO, is a major factor limiting the postharvest quality and shelf life of horticultural produce. Potato tubers are particularly prone to enzymatic browning after cutting, making it an ideal material for investigating regulatory mechanisms underlying this process. Although transcriptional regulation of *PPO* has been widely studied, the post-translational mechanisms that modulate its activity remain largely unexplored. In this study, through transcriptomic comparisons between browning-sensitive and browning-resistant potato cultivars, combined with physiological assessments of IAA-mediated browning inhibition, we identified the auxin-responsive gene *StSAUR31* as a key regulator of PPO activity and enzymatic browning in potatoes (Fig. 1). Subsequent analyses integrating biochemical assays, proteomics, protein–protein interaction studies, and subcellular localization experiments revealed the molecular mechanisms by which *StSAUR31* modulated PPO activity at the protein level. These findings broaden our understanding of the crosstalk between auxin signaling and enzymatic browning regulation, offering new mechanistic perspectives for enhancing postharvest quality in horticultural crops.

Plant hormones, particularly auxin, play crucial roles in maintaining postharvest quality by modulating diverse physiological and metabolic processes. Our study demonstrated that IAA treatment inhibited PPO activity in potatoes (Fig. 1). However, previous studies have shown that auxins, including IAA, 2,4-D, NAA, and IBA, enhanced PPO activity during adventitious root formation and callus development in cuttings [27–29], which appears contradictory to our findings. These observations suggested that the regulation of PPO activity by auxin was not uniform but instead exhibited significant diversity. For example, during active growth and organogenesis, PPO participates in the generation of IAA-phenolic complexes that function as cofactors for root initiation and cellular differentiation [30]. Thus, PPO upregulation is adaptive and beneficial for tissue regeneration in these contexts. In contrast, in wounded fruits and vegetables, the elevated PPO activity serves as a primary driver of enzymatic browning [1]. Previous studies have reported that applying IAA to intact mango and royal dates reduced PPO activity and alleviated peel browning [14, 15]. Consistent with these observations, our study showed that applying IAA to intact potato tubers significantly inhibited PPO activity, whereas direct application to fresh-cut potato slices failed to suppress browning (Fig. 1E; Fig. S3). These results suggested that auxin-mediated inhibition of browning may require regulatory processes established before tissue damage. After cutting, PPO is rapidly activated due to cellular compartment disruption, and post-cutting IAA treatment may be insufficient to counteract this immediate enzymatic activation. In contrast, pre-cutting IAA treatment may establish a physiological state that facilitates the regulation of enzymatic browning after wounding. Collectively, these findings indicated that auxin-mediated regulation of PPO activity may be context-dependent and influenced by the developmental and physiological status of the tissue.

As downstream components of auxin signaling, *SAURs* are rapidly induced by IAA and function as key mediators of auxin-regulated growth and development [21, 31–34]. *SAUR* family proteins have been reported to modulate auxin biosynthesis or distribution in different plant species. For example, rice *OsSAUR45* negatively regulated auxin levels by repressing *OsYUCCA* and *OsPIN* gene expression [33]. Several *Arabidopsis* and maize *SAURs* (e.g. *AtSAUR19-24, AtSAUR63, ZmSAUR2*) promoted auxin accumulation by enhancing auxin transport [34–36]. Our results showed that overexpression of *StSAUR31* increased endogenous IAA levels, accompanied by elevated expression of *StYUCCA6*, *StYUCCA8*, and *StYUCCA10* (Fig. S6). Despite extensive characterization of *SAURs* in growth and developmental processes, their roles in enzymatic browning have not been explored. Browning is primarily triggered by mechanical wounding, a form of abiotic stress that disrupts cellular compartmentalization and induces oxidative responses [1]. The responsiveness of *SAUR* genes to wounding and biotic stresses suggested their potential involvement in the physiological regulation of stress adaptation [22, 23]. Here, we identified *StSAUR31* as an auxin-inducible gene that exhibited higher expression in browning-resistant cultivar than in browning-susceptible ones (Fig. 1; Table 1). Overexpression of *StSAUR31* suppressed tuber browning, at least in part, through direct interaction with StuPPO1 and partial alteration of its subcellular localization (Figs 4 and 5). These findings revealed a novel post-translational regulatory mechanism by which *StSAUR31* connected auxin signaling to PPO activity.

PPO is synthesized in the cytosol and must be imported into plastids for activation [8]. Therefore, correct and efficient plastid targeting is essential for its enzymatic function. Increasing evidence suggests that plastid-targeted proteins can be regulated prior to import, thereby affecting their subcellular localization and enzymatic function. Such regulation occurred at multiple levels. For example, in *Medicago truncatula*, the precursor of glutamine synthetase (GS2) specifically accumulated at the plastid surface, resulting in reduced levels of active GS2 within plastids [9]. In addition, chemical modifications of plastid proteins have been shown to influence the efficiency of plastid import [10]. Accumulating studies indicate that protein–protein interactions play a critical role in regulating protein subcellular localization [37, 38]. In this study, we demonstrated that StSAUR31 directly interacted with StuPPO1 (Fig. 4). Using transient expression assays in tobacco leaves and potato protoplasts, we further showed that StSAUR31 partially altered the subcellular localization of StuPPO1, thereby reducing its plastid accumulation (Figs 5 and S8). Notably, auxin enhanced the interaction between StSAUR31 and StuPPO1, further promoting this process. PPO is predominantly localized to chloroplasts in leaf tissues, whereas it primarily exists in amyloplasts in potato tubers [6]. Although the physiological contexts of leaf mesophyll cells differed from those of tuber cells, the core components of the plastid protein import machinery, including the TOC/TIC translocon complexes, were conserved across diverse plastid types [39, 40]. Notably, *in vitro* import assays have demonstrated that chloroplast-specific precursor proteins can be imported into non-green plastids, and vice versa [40–43]. Based on this evidence, we speculated that the regulatory mechanism by which StSAUR31 modulated StuPPO1 localization may not be restricted to chloroplasts, but could also be applicable in amyloplasts. These findings provided a plausible mechanistic basis for the regulatory role of StSAUR31 in PPO-mediated browning in potato tubers.

**Figure 6:**
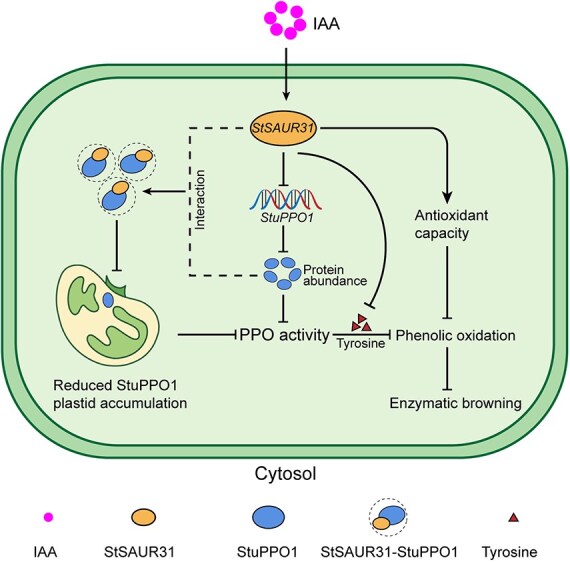
Proposed model for *StSAUR31*-mediated browning regulation in potatoes. Upon exogenous IAA treatment, *StSAUR31* expression was induced, which suppressed enzymatic browning through multiple mechanisms. StSAUR31 directly interacted with StuPPO1 and partially altered its subcellular localization, reducing StuPPO1 accumulation in plastids. Meanwhile, *StSAUR31* downregulated the expression of *StuPPO1*, leading to reduced StuPPO1 protein abundance. These two factors collectively contributed to the decrease in PPO activity. Furthermore, *StSAUR31* reduced endogenous free tyrosine content and enhanced the antioxidant capacity. These mechanisms collectively suppressed PPO-mediated phenolic oxidation and ultimately inhibited enzymatic browning. Arrows denote activation; lines with flat ends represent inhibition; dashed lines indicate protein–protein interaction.

Our results indicated that *StSAUR31* regulated PPO activity not only by affecting the subcellular localization of *StuPPO1* but also by negatively regulating *StuPPO1* transcription (Fig. S11A). *StSAUR31* overexpression reduced *StuPPO1* transcript levels by approximately 50%, which provided an explanation for the observed decrease in StuPPO1 protein abundance (Fig. 3C). This reduction in StuPPO1 protein levels represented an important contributor to the decreased PPO activity. Meanwhile, *StSAUR31* also affected the expression of *StuPPO2* and *StuPPO3* (Fig. S11B and C), suggesting that *StSAUR31* exerted broad transcriptional control over the principal PPO isoforms. Notably, our recent study identified StODO1 as a positive regulator of *StuPPO1–3* expression [25]. In the present study, *StSAUR31* overexpression repressed *StODO1* levels, while its knockout upregulated *StODO1* expression (Fig. S12). These results suggested that *StSAUR31* may indirectly modulate the transcriptional profiles of *StuPPO1–3* through StODO1-mediated regulatory pathway. However, Y2H assays revealed that StSAUR31 interacted specifically with StuPPO1 but not with StuPPO2 or StuPPO3 (Figs 4 and S13). This selectivity may be explained by the differential wound responsiveness of *PPO* isoforms. Our previous study demonstrated that *StuPPO1* was the most strongly wound-induced *PPO* gene compared to *StuPPO2* and *StuPPO3* [25]. Notably, it showed a cultivar-specific pattern, with *StuPPO1* induction substantially lower in ‘K13’ than in ‘K4’ at 3 d after cutting [25]. Therefore, the specific interaction between StSAUR31 and StuPPO1 likely reflected a regulatory strategy focused on the major cutting-responsive isoform, enabling timely modulation of PPO activity and enzymatic browning. While our Y2H assay indicated no direct interaction between StSAUR31 and StuPPO2/StuPPO3, the possibility of indirect regulation through intermediate proteins cannot be excluded and warrants future investigation.

Tyrosine serves as an important phenolic substrate in potato browning, and its content directly influences the initiation and extent of the browning reaction [26]. Our results showed that the expression level of *StSAUR31* was significantly associated with the browning phenotype in potato. Prior to the onset of browning (samples collected immediately after cutting, at 0 d), tyrosine content was significantly reduced in *StSAUR31* overexpression lines but markedly increased in knockout lines (Fig. S14A). These findings suggested that *StSAUR31* may participate in the early-stage regulation of browning by modulating the basal biosynthesis or metabolism of tyrosine. Free tyrosine levels in potato tubers are predominantly derived from protein catalyzed by endogenous proteases, which can be regulated by protease inhibitors [26, 44]. Previous studies demonstrated that overexpression of protease inhibitor genes suppressed protein degradation, thereby reducing free tyrosine content and alleviating browning in potatoes [26]. Our proteomic analysis revealed that the abundance of three protease inhibitors (M1AJ83, J7EQ13, M1AMZ1) was significantly downregulated in *stsaur31* knockout lines compared with WT (Table S3). This downregulation likely promoted protein hydrolysis, thereby increasing the accumulation of free tyrosine and potentially contributing to browning. Previous studies have shown that *SAURs* participate in redox regulation by modulating the ROS scavenging system [45, 46]. In the present study, *StSAUR31* overexpression significantly improved DPPH radical scavenging capacity (Fig. S14B), which could help maintain cellular redox homeostasis and thereby alleviating enzymatic browning. However, compared with the substantial reduction in tyrosine content (approximately 46.6% decrease), the changes in DPPH radical scavenging capacity were relatively modest, showing only a 27.8% increase in *StSAUR31* overexpression lines. These results suggested that alterations in tyrosine availability may have a more pronounced impact on browning than the observed changes in antioxidant capacity. Nevertheless, the integration of these regulatory mechanisms likely determines the overall browning phenotype. Together, our study reveals that *StSAUR31* functions as a regulatory hub that integrates PPO activity, tyrosine metabolism, and antioxidant defense to collectively regulate browning in potatoes (Fig. 6). Future investigations tracking the dynamics of these parameters during the browning process will help clarify their relative contributions to *StSAUR31*-mediated browning inhibition.

## Conclusion

In summary, we identified an auxin-inducible gene, *StSAUR31*, which exhibited higher expression levels in browning-resistant potato cultivars than in browning-susceptible ones. Functional analyses demonstrated that *StSAUR31* negatively regulated enzymatic browning in potatoes. Mechanistically, StSAUR31 not only directly interacted with StuPPO1 to reduce its plastid accumulation, but also downregulated *StuPPO1* transcription to decrease protein abundance. Additionally, *StSAUR31* reduced free tyrosine content and enhanced antioxidant capacity, which further alleviated PPO-mediated enzymatic browning. Collectively, these findings revealed a novel regulatory mechanism linking auxin signaling to PPO-mediated enzymatic browning, providing new insights into the hormonal regulation of postharvest quality in horticultural crops.

## Materials and methods

### Plant material

Transcriptome data of the browning-sensitive cultivar ‘K4’ and the browning-resistant cultivar ‘K13’ at 0 d and 3 d after cutting were obtained from our previously published studies [25, 26]. In the present study, *Solanum tuberosum* L. cultivar ‘Desiree’ was used as the genetic background for both overexpression and knockout experiments due to its stable and efficient genetic transformation system [4, 26, 47]. Potato plantlets were grown on MS basal medium containing 2.0% sucrose and 0.8% agar, under controlled growth conditions of 16 h light (23°C) and 8 h dark (21°C) with 65 ± 5% relative humidity. After 3–4 weeks of growth, plantlets were used for genetic transformation.

To maintain genetic background consistency, exogenous IAA treatment was also performed using tubers of the same cultivar (‘Desiree’). ‘K4’ and ‘Netherland 15’ are widely cultivated in China and are susceptible to browning. Therefore, these two cultivars were used to validate the generality of IAA-induced browning inhibition. These two cultivars were harvested in Heilongjiang and Shandong Province, respectively. After harvesting, potato tubers were immediately transported to the laboratory and stored at 4°C until use.

### IAA treatment

IAA (≥99% purity) was purchased from Biotopped Technology Co., Ltd. Potato cultivar ‘Desiree’ was used for exogenous IAA treatment. Uniform, disease-free tubers were selected, washed, and surface-sterilized with 200 μl/l sodium hypochlorite. For post-cutting treatment, fresh-cut potato slices (2–3 mm thick) were soaked in 0 (control), 1, 5, 25, and 100 mg/l IAA solutions for 10 min, drained and packaged, with 15 slices per bag and three bags per treatment. Samples were stored at 4°C. For pre-cutting treatment, in preliminary experiments, whole tubers were treated with IAA solutions (0, 1, 5, 25, and 100 mg/l) for 10 min and then held at 25°C for 0–72 h to determine the optimal treatment combination. Based on the preliminary results, 5 mg/l IAA followed by 48 h holding at 25°C was identified as the optimal treatment condition. The treated samples (0 and 5 mg/l IAA) were cut into slices and packed into low-density polyethylene (LDPE) bags, with 15 slices per bag and eighteen bags for each treatment. Samples were stored at 4°C for 0, 1, 2, 3, 4, and 5 d, respectively. At each time point, half of the slices were used for visual quality assessment, and the remaining were frozen in liquid nitrogen for further analysis. Another set of experiments was conducted under the same treatment conditions, in which samples were collected at 0, 12, 24, and 48 h during the holding period to determine PPO activity and *SAURs* gene expression.

### Visual quality evaluation and color measurement

The overall visual quality and visual browning were assessed following Feng *et al*. [48]. Overall visual quality was rated on a 9-point scale: 9 indicates excellent (fresh appearance); 7, good; 3, poor; and 1, unusable. A visual quality score below 6 was considered as loss of marketability. Visual browning was assessed using a 5-point scale, where 1 represents no browning, 3 represents moderate browning, and 5 represents severe browning. In addition, the lightness (L^*^) value was recorded using a colorimeter. For each treatment, ten potato slices were randomly selected, and the L^*^ values were measured at the central surface. The reported L^*^ values represented the mean of ten measurements.

### Gene expression analysis

Total RNA was isolated and reverse-transcribed into cDNA using commercial kits (Vazyme, China). The resulting cDNA served as templates for quantitative real-time PCR (qRT-PCR), which was conducted on a CFX96 Real-Time PCR System (Bio-Rad). Each 20 μl reaction mixture comprised 1 μl of cDNA, 10 μl of Mix, 8.2 μl of ddH_2_O, and 0.4 μl of each gene-specific primer. The amplification protocol and reaction parameters adhered to the manufacturer’s guidelines (Q321-02, Vazyme). All primer sequences can be found in Table S4.

### Generation of *StSAUR31* overexpression and knockout lines

The coding sequence of *StSAUR31* (*PGSC0003DMG400030231*, *Soltu.DM.06G014110.1*) was amplified and inserted into ph7lic-C-HA vector, and introduced into *Agrobacterium tumefaciens* strain AGL1 + virG. For CRISPR/Cas9-mediated knockout of StSAUR31, two guide RNA sequences were identified using CRISPR-P 2.0, named sgRNA-1 (g1: GAAATGATAGGCATCCCAAAGGG) and sgRNA-2 (g2: CCAGTATTGGTAGGTCATGATGG). The corresponding oligonucleotides were synthesized by Sangon Biotech and ligated into the pCAMBIA1300-based CRISPR/Cas9 vector downstream of the *Arabidopsis* U6-26 promoter (AtU6–26p). Two independent knockout constructs were generated and separately transformed into AGL1 + virG for potato transformation. The transformed *Agrobacterium* cultures were grown in LB medium at 28°C for 48 h, centrifuged and resuspended in liquid MS medium, and adjusted to an OD_600_ value of 0.6–0.8 for infection. Potato transformation was performed following Li *et al*. [49], with slight modifications. Internodal stem segments from 3–4-week-old ‘Desiree’ plantlets were precultured on MS medium for 2 d, immersed in the infection solution for 5 min, then cocultivated in the dark at 28°C for 2 d. Thereafter, explants were transferred to callus induction medium and shoot induction medium. Transformed shoots were regenerated on selective medium supplemented with 50 mg/l kanamycin. The expression levels of *StSAUR31* were determined to identify positive transformants. For knockout lines, genomic DNA was extracted from regenerated shoots using a commercial kit (Vazyme, China). The successful integration of T-DNA was first verified by PCR with Cas9-specific primers. Then, the target regions of *StSAUR31* were amplified, and the PCR products were subjected to next-generation sequencing to identify homozygous mutations. Two overexpression lines showing the highest *StSAUR31* transcript levels (*OX1* and *OX2*) and two homozygous knockout lines (*ko1* and *ko2*) were chosen. Ten plantlets per line were grown in a greenhouse (16 h light at 23°C and 8 h dark at 21°C). Tubers were harvested four months after transplanting.

After harvest, potato tubers with uniform size and shape and free from visible defects or disease were selected. Tubers were cut into slices, placed into LDPE bags and stored at 4°C for 0 (samples collected immediately after cutting), 1, 2, and 3 d (three bags per day, 15 slices per bag). The overall visual quality and L^*^ value were measured. To evaluate the inherent effect of *StSAUR31* on basal metabolism, fresh-cut potato slices of 0 d (samples collected immediately after cutting, no browning occurred) were ground into powder, and stored at −80°C for subsequent analysis of PPO activity, tyrosine content, and antioxidant capacity.

### P‌PO activity assays

PPO activity was measured following Feng *et al*. [48] with minor modifications. Potato powder samples (1.0 g) were mixed with 3.5 ml phosphate buffer (0.1 M, pH 6.8), swirled and centrifuged at 12 000 × *g* for 15 min at 4°C to get the crude enzyme. The reaction mixture consisted of 1.2 ml PBS, 0.8 ml of 0.02 mol/l catechol solution, and 0.5 ml crude enzyme. The absorbance was recorded at 410 nm. One unit (U) of PPO activity was defined as a 0.01 increase in absorbance per minute.

### Proteomic analysis

Protein extraction was performed using powdered samples of WT and *stsaur31* mutants, with three biological replicates per genotype. Total proteins were extracted using the phenol/methanolic ammonium acetate method [50]. The protein pellets were rinsed with prechilled methanol and acetone, dissolved in 8 M urea, and their concentrations were measured using a BCA assay kit (Beyotime Biotechnology, China). For each biological replicate, 0.2 mg total protein was digested with 2 μg trypsin. The obtained peptides were labeled with TMT reagents (Thermo Scientific, Rockford, IL, USA). TMT-labeled peptides were then fractionated and ionized by a nanospray ionization (NSI) source and analyzed on an Orbitrap Exploris™ 480 mass spectrometer. MS1 was performed over 400–1200 m/z with a resolving power of 60 000. MS2 were collected at a resolution of 15 000 with the scan range of 110 m/z. Fragmentation was performed using higher-energy collisional dissociation at a normalized collision energy of 35%. Data-dependent acquisition was operated in a cycle time-based mode with a 1.0 s cycle time, selecting precursor ions for fragmentation in order of decreasing intensity within each cycle. Proteins were identified by searching against the potato database (https://spuddb.uga.edu/). Proteins with fold change ≥ 1.3 and *P* ≤ 0.05 were defined as differentially accumulated proteins. Functional annotation of the identified DAPs was performed using the UniProt database. Subsequently, GO enrichment analysis was conducted using Oebiotech cloud platform (https://cloud.oebiotech.com/) to elucidate enriched pathways.

### IAA content assay

The IAA content was determined based on a previous study with minor modifications [51]. Potato powders (0.2 g) were weighed into precooled centrifuged tubes containing 1.0 ml of 80% methanol, extracted at 4°C for 12 h, and centrifuged at 8000 × *g* for 10 min to obtain the supernatant. The remaining residue was extracted with 0.3 ml methanol, followed by centrifugation. The supernatant from the above two steps was combined and evaporated by nitrogen (4°C) to obtain a non-organic phase. A volume of 0.3 ml petroleum ether was added for discoloration, and the upper ether phase was subsequently discarded. Meanwhile, the lower layer was dried with nitrogen, followed by dissolution in 0.3 ml mobile phase. The solution was then filtered through an organic membrane (0.45 μm) before HPLC analysis. IAA content was measured using a C18 reversed-phase column (Agilent, USA). The mobile phase mixture consisted of 60% water and 40% methanol, containing 0.6% acetic acid. A 10 μl sample was introduced into the system with a flow rate of 0.8 ml/min. Chromatographic separation was carried out at 35°C, and the excitation wavelength, as well as the emission wavelength, were set at 275 and 345 nm, respectively.

### Y2H assay

Y2H experiment was carried out based on a previous protocol [52]. The coding sequences of *StuPPO1–3* and *StSAUR31* were cloned and transformed into pGADT7 and pGBKT7 vectors, respectively. The recombinant plasmids were cotransformed into Y2HGold competent cells, and cultivated on SD/-Trp/-Leu solid medium (Coolaber, Beijing, China) at 30°C for 2 d. The positive transformants were lined and cultured on SD/-Trp/-Leu/-His/-Ade solid medium (Coolaber, Beijing, China) at 30°C for 3 d. Besides, the yeast colonies of StSAUR31-pGBD and StuPPO1-pGAD, cultivated on SD/-T/-L solid medium, were chosen and cultured in SD/-T/-L liquid medium until reaching an OD_600_ value of 0.4. After serial dilution, 10 μl of each yeast suspension was inoculated on SD/-T/-L/-H/-A solid medium (containing x-α-gal) with 0 μM and 10 μM IAA.

### LCI assay

The coding sequences of *StSAUR31* and *StuPPO1* were cloned into pCAMBIA1300-cLUC and pCAMBIA1300-nLUC vectors, mixed and transiently expressed in *N. benthamiana* leaves following the method described previously [53]. For IAA treatment, 100 μM IAA, a concentration previously used in *N. benthamiana* transient expression assays [54], was infiltrated into tobacco leaves at 12 h after agroinfiltration. At 48 h after agroinfiltration, 1 mM of D-luciferin solution (Solarbio, China) was sprayed on the leaves, and incubated in darkness for 2 min. Luminescence was then captured using an *in vivo* imaging system.

### BiFC assay

The coding sequences of *StuPPO1* and *StSAUR31* were inserted into pSPYNE-35S and pSPYCE-35S to generate StuPPO1-YFP^N^ and StSAUR31-YFP^C^ constructs, respectively, and transformed into GV3101. The *A. tumefaciens* colonies carrying StuPPO1-YFP^N^ and StSAUR31-YFP^C^, and empty YFP^N^ and YFP^C^ (as control), were cotransformed into the *N. benthamiana* leaves, respectively. IAA treatment was performed following the same procedure as described for the LCI assay. Fluorescence signals were observed using a two-photon laser confocal microscope (ZEISS, Germany).

### Subcellular localization assay

The coding sequence of *StSAUR31* was cloned into the pRI-GFP vector to generate *StSAUR31*-GFP. The StuPPO1-RFP construct was generated by recombination of the *StuPPO1* coding sequence into HS-mscarlet3-RFP. All the recombinant constructs were then transformed into GV3101. The *A. tumefaciens* colonies carrying StSAUR31-GFP and StuPPO1-RFP, and empty GFP and StuPPO1-RFP (as control) were cotransformed into the *N. benthamiana* leaves, respectively. Then the fluorescence was detected as described for BiFC.

### Isolation of total and chloroplast proteins and western blot analysis

Tobacco leaves co-expressing empty GFP + StuPPO1-RFP (as control), StSAUR31-GFP + StuPPO1-RFP (without IAA treatment), and StSAUR31-GFP + StuPPO1-RFP (IAA treatment) were used for total and chloroplast protein extraction. Chloroplasts were isolated following the procedure of Sun *et al.* [55]. Approximately 1 g of leaf tissue was homogenized in 1× chloroplast isolation buffer (CIB) on ice and centrifuged at 3000 × *g* at 4°C for 5 min. The obtained pellet was resuspended in 1× CIB buffer, and intact chloroplasts were purified using 20%/40%/80% step Percoll gradient [56]. The purified chloroplasts were then resuspended in 1× CIB buffer and used for protein extraction. Total and chloroplast proteins were extracted using a commercial kit (CW0885M, CWBIO, China). A custom rabbit polyclonal anti-StuPPO1 antibody was generated by Sangon Biotech and used at a dilution of 1:1000 as the primary antibody. Mouse monoclonal anti-Actin (Abbkine, ABL1050) and anti-Rubisco (Biodragon, B1485) antibodies were used at 1:2000 as loading controls for total and chloroplast proteins, respectively. HRP-conjugated goat anti-rabbit IgG (Abbkine, A21020) and goat anti-mouse IgG (Abways, AB0102) were used at 1:10 000 dilution as secondary antibodies.

### Protoplast isolation and transient transformation

Potato leaf protoplasts were isolated following the method of Jiang *et al*. [57]. Cellulase R10 and macerozyme R10 were purchased from Yakult Honsha (Tokyo, Japan), while all other reagents were obtained from Sigma-Aldrich (St. Louis, MO, USA). Young leaves from 4-week-old ‘Desiree’ and *StSAUR31*-overexpressing lines were used for protoplast isolation. The upper portions of leaves containing prominent veins were removed. Leaf tissues were gently peeled using adhesive tape, cut into small pieces, and immediately incubated in 12 mL enzyme solution containing 20 mM 2-(N-morpholino) ethanesulfonic acid (MES, pH 5.7), 20 mM KCl, 0.3 M mannitol, 1.5% cellulase R10, 0.3% macerozyme R10, 10 mM CaCl_2_, and 0.1% bovine serum albumin (BSA). The samples were vacuum infiltrated at approximately −0.09 MPa for 30 min and then incubated on a shaker (26°C, 45 rpm) for 3 h in the dark. After digestion, 10 ml of W5 solution (150 mM NaCl, 125 mM CaCl_2_, 5 mM KCl, and 2 mM MES) was added to the mixture and swirled gently. The suspension was filtered through a 200-mesh cell filter into centrifuge tube and centrifuged. The resulting pellet was resuspended in 10 ml W5 solution, incubated on ice for 20 min, followed by centrifugation at 100 × *g* for 2 min. The protoplasts were finally resuspended in 1 ml MMG solution (4 mM MES, pH 5.7; 0.4 M mannitol, and 15 mM MgCl_2_). For transient expression assays, 100 μl of protoplast suspension was mixed with 10 μg of StuPPO1-GFP plasmid, followed by the addition of 110 μl PEG solution (40% PEG4000, 150 mM mannitol, and 100 mM CaCl_2_). The mixture was gently flicked to mix and incubated at 22°C for 20 min. Subsequently, 440 μl W5 solution was added to terminate the transformation, and protoplasts were collected by centrifugation at 100 × *g* for 2 min. The pellet was resuspended in 1 ml W5 solution and incubated at 22°C in the dark for 16–18 h before fluorescence observation.

### Statistical analysis

All experiments were conducted in three replications, using a completely randomized factorial design, and data were presented as mean ± SD. All statistical analyses were conducted with GraphPad Prism 9.0 (GraphPad Software, San Diego, California, USA) and SPSS 17.0 Statistical Software Program (SPSS Inc., Chicago, IL, USA). Statistical significance was determined using one-way ANOVA or Student’s *t*-test.

## Supplementary Material

Web_Material_uhag115

## Data Availability

All data is available within the manuscript and its supporting materials.
