# Supplementary material for: StSAUR31 functions as a negative regulator of enzymatic browning in potato (Solanum tuberosum L.)
Source: Hortic Res. 2026 Apr 10;13(7):uhag115. doi: 10.1093/hr/uhag115 (PMC13326752; doi:10.1093/hr/uhag115)
Supplement: Web_Material_uhag115 [file web_material_uhag115.zip › Supplementary Figure.docx]

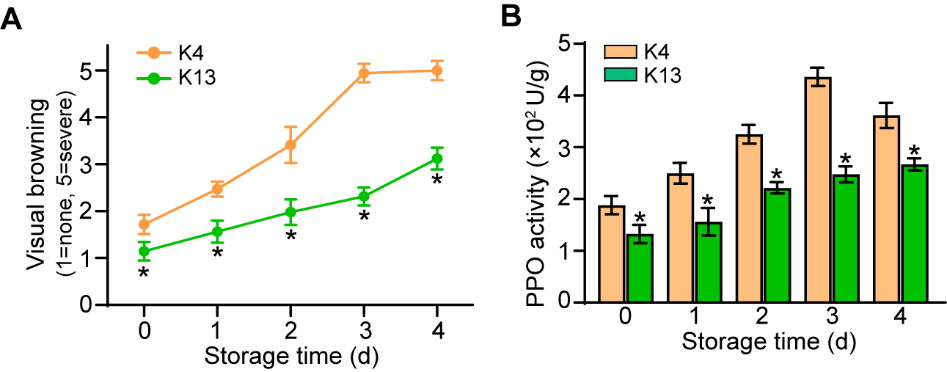


**Fig. S1.** Changes of visual browning (A) and PPO activity (B) in ‘K4’ and ‘K13’ tubers following fresh-cut treatment. The asterisks indicate significant differences (**P* < 0.05) as determined by Student’s *t*-test.

**
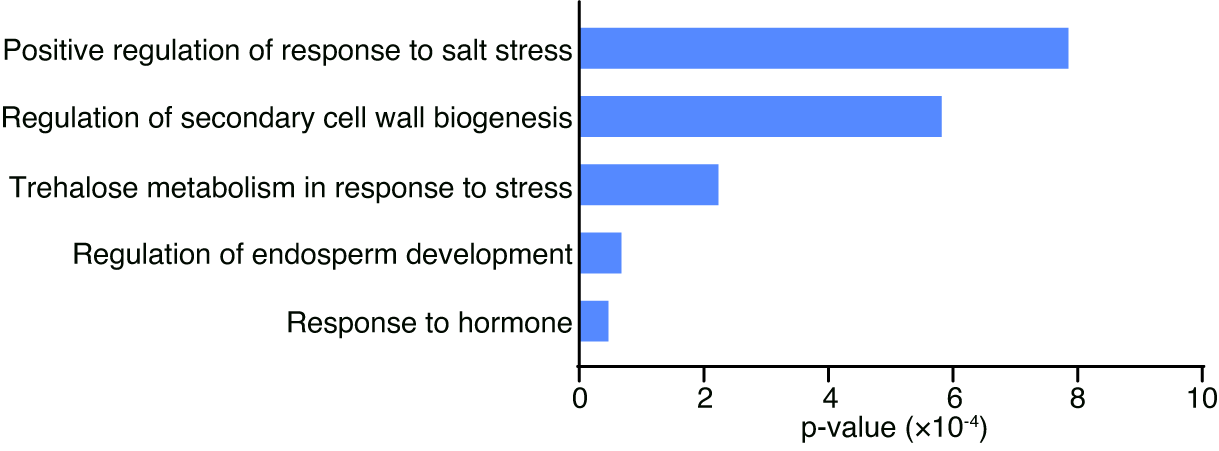
**

**Fig. S2.** Gene Ontology (GO) enrichment of differentially expressed genes (DEGs) of ‘K4’ and ‘K13’ tubers following cutting treatment.


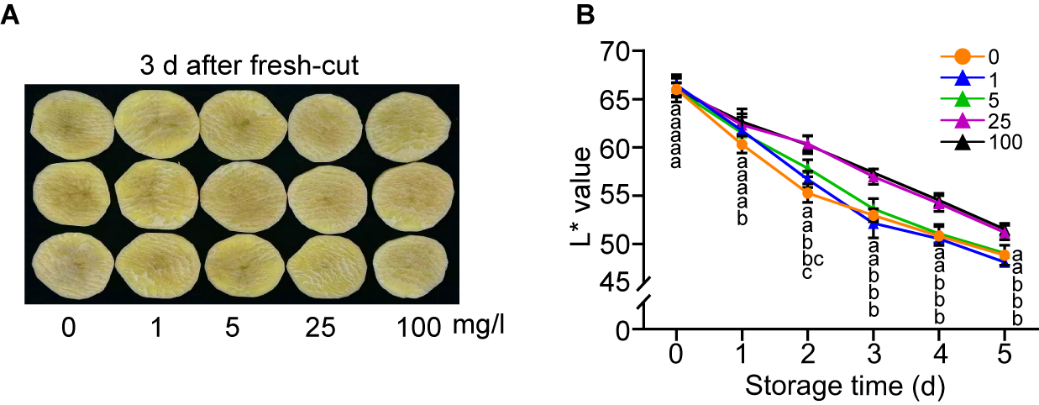


**Fig. S3.** Effects of post-cutting IAA treatment on the browning of potato slices. (A) Visual browning. (B) L* values. Different letters indicate significant differences among samples (*P* < 0.05, one-way ANOVA).


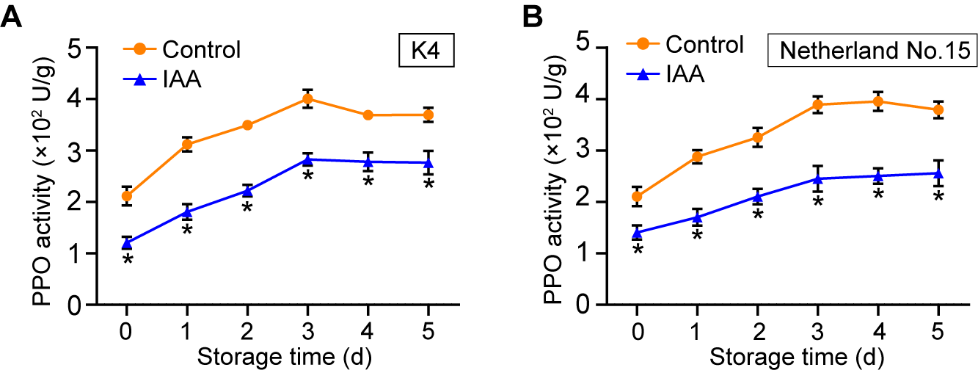


**Fig. S4.** PPO activity of ‘K4’ (A) and ‘Netherland No. 15’ (B) potato cultivars treated with 5 mg/l IAA (pre-cutting treatment). The asterisks indicate significant differences (**P* < 0.05) as determined by Student’s *t*-test.


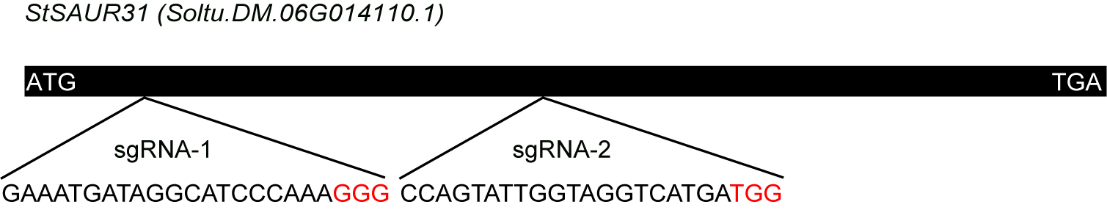


**Fig. S5.** Schematic representation of CRISPR/Cas9 constructs used to generate *stsaur31* knockout lines. Two target sites (sgRNA-1 and sgRNA-2) were designed at distinct positions within the *StSAUR31* coding sequence, with PAM motifs highlighted in red.


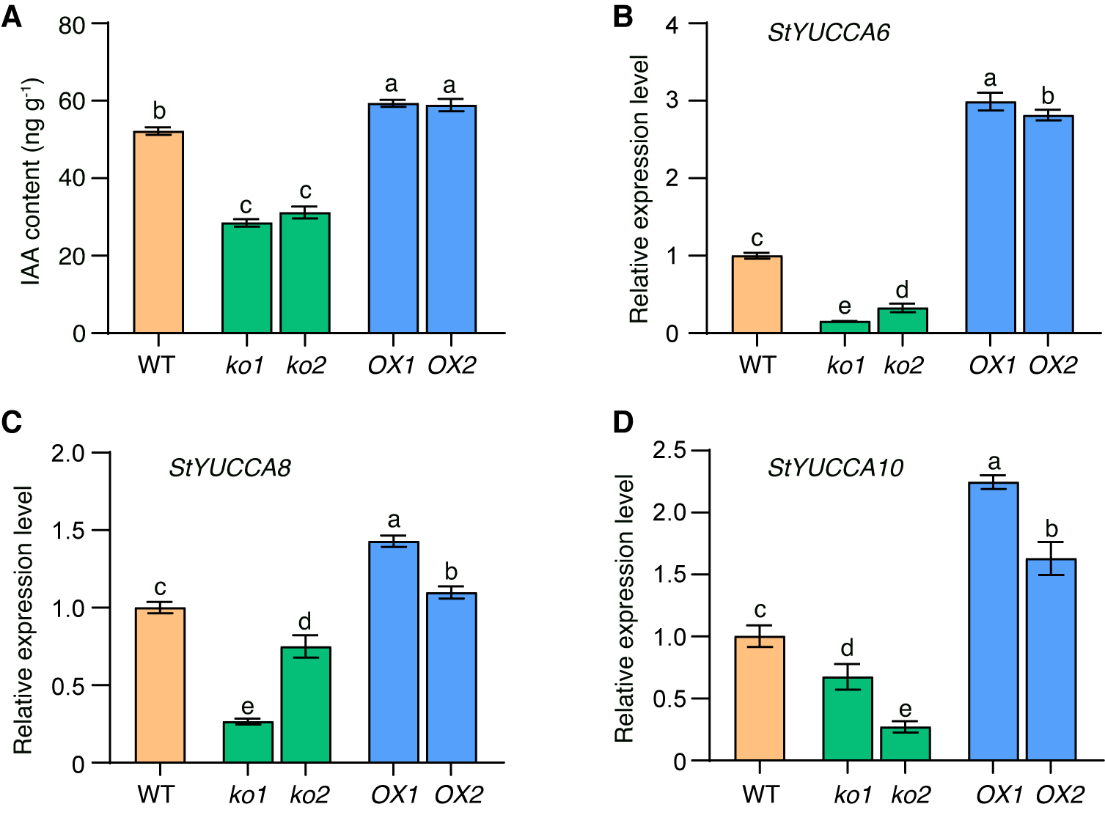


**Fig. S6.** *StSAUR31* modulates free IAA levels. (A) IAA content in WT and *StSAUR31* transgenic tubers. (B–D) Relative expression levels of auxin biosynthesis genes (*StYUCCA6*, *StYUCCA8*, and *StYUCCA10*). Data are presented as mean ± SD. Different letters indicate significant differences among samples (*P* < 0.05, one-way ANOVA).


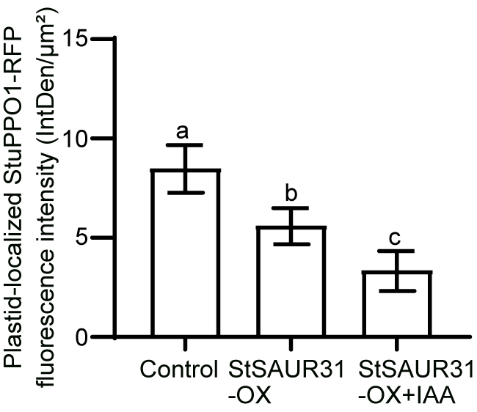


**Fig. S7.** Quantification of plastid-localized StuPPO1-RFP fluorescence intensity in control and *StSAUR31*-overexpressing tobacco leaves. Fluorescence intensity was measured using ImageJ. Data represent the mean ± SD of three independent biological replicates. Different letters indicate significant differences at *P* < 0.05 (one-way ANOVA).


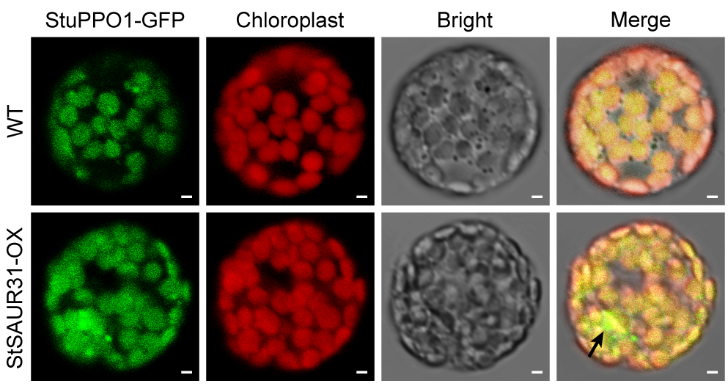


**Fig. S8.** Transient expression of StuPPO1-GFP in protoplasts of potato leaves from wild-type (WT) and *StSAUR31*-overexpressing (*OX*) lines. Scale bar, 2 μm. Arrows indicate GFP signals localized outside the chloroplasts.


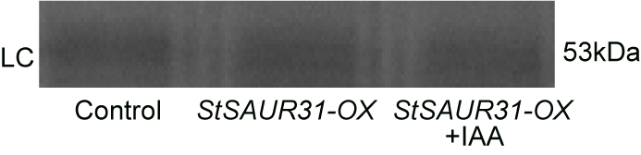


**Fig. S9.** Verification of Rubisco as an internal reference protein for chloroplast proteins. The Coomassie-stained protein SDS-PAGE gel, showing consistent Rubisco abundance across samples.


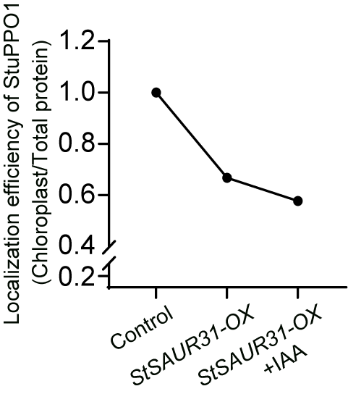


**Fig. S10.** Quantification of the chloroplast localization efficiency of StuPPO1. The chloroplast localization efficiency of StuPPO1 was defined as the ratio of chloroplast-localized StuPPO1 to total StuPPO1 protein. The localization efficiency in the control was set to 1.


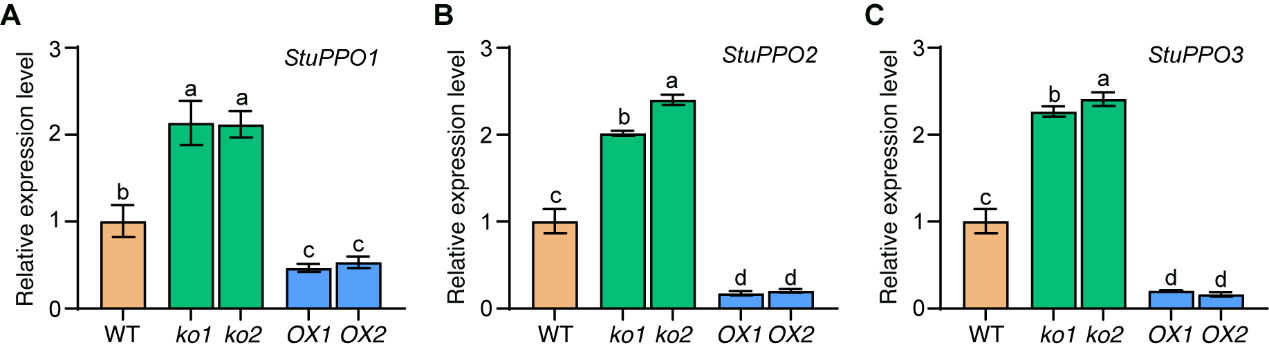


**Fig. S11.** Relative expression levels of *StuPPO1-3* in WT and *StSAUR31* transgenic tubers. Different letters indicate significant differences among samples (*P* < 0.05, one-way ANOVA).


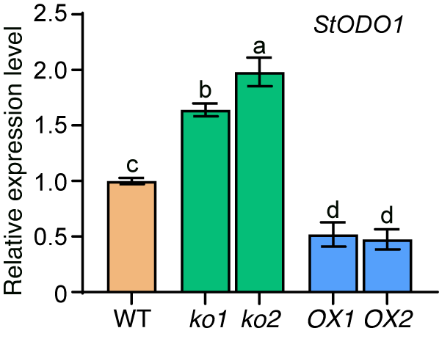


**Fig. S12.** Relative expression levels of *StODO1* in WT and *StSAUR31* transgenic tubers. Different letters indicate significant differences among samples (*P* < 0.05, one-way ANOVA).


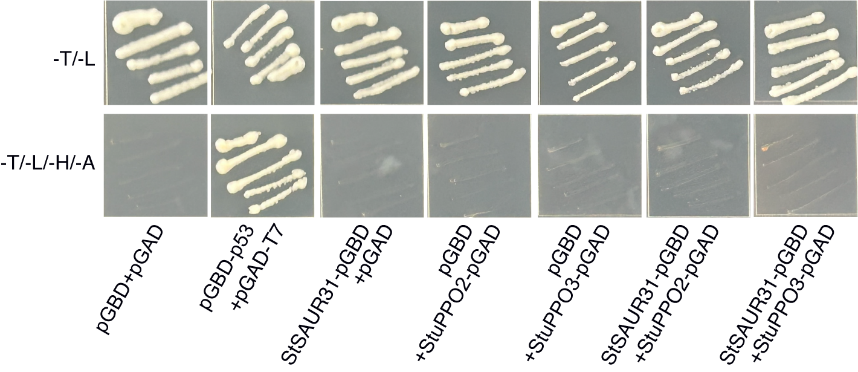


**Fig. S13.** Yeast two-hybrid (Y2H) analysis of the interactions between StSAUR31 and StuPPO2-3. The combination of pGBD-p53 and pGAD-T7 served as a positive control.


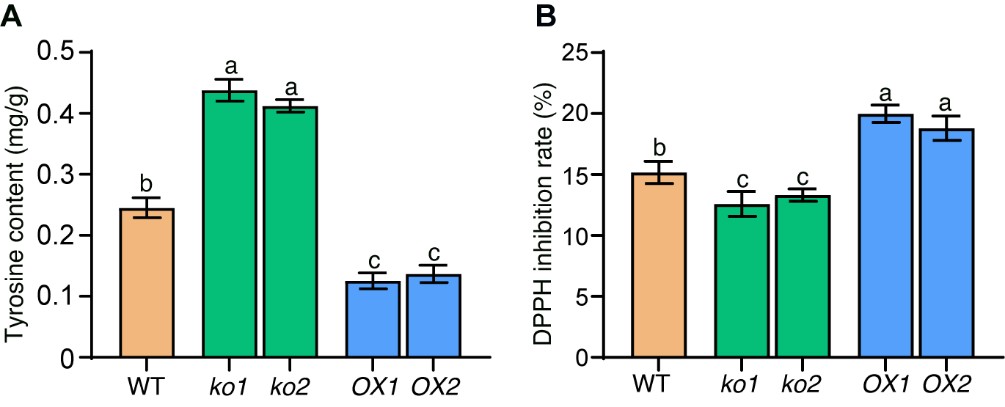


**Fig. S14.** Free tyrosine content (A) and DPPH inhibition rate (B) in WT and *StSAUR31* transgenic tubers at day 0 (samples collected immediately after cutting). Different letters indicate significant differences among samples (*P* < 0.05, one-way ANOVA).
